# Colonisation Factor CD0873, an Attractive Oral Vaccine Candidate against *Clostridioides difficile*

**DOI:** 10.3390/microorganisms9020306

**Published:** 2021-02-02

**Authors:** Cansu Karyal, Jaime Hughes, Michelle L. Kelly, Jeni C. Luckett, Philip V. Kaye, Alan Cockayne, Nigel P. Minton, Ruth Griffin

**Affiliations:** 1 Synthetic Biology Research Centre, The University of Nottingham Biodiscovery Institute, University Park, Nottingham NG7 2RD, UK; cansu.karyal@nottingham.ac.uk (C.K.); jaime.hughes@nottingham.ac.uk (J.H.); michelle.kelly@nottingham.ac.uk (M.L.K.); alan.cockayne1@nottingham.ac.uk (A.C.); nigel.minton@nottingham.ac.uk (N.P.M.); 2 The University of Nottingham Biodiscovery Institute, University Park, Nottingham NG7 2RD, UK; jeni.luckett@nottingham.ac.uk; 3 Department of Histopathology, Queen’s Medical Centre, Nottingham University Hospitals NHS Trust, Nottingham NG7 2UH, UK; philip.kaye@nuh.nhs.uk; 4 National Institute for Health Research (NIHR) Nottingham Biomedical Research Centre (BRC), Nottingham NG7 2UH, UK

**Keywords:** mucosal immunity, sIgA, oral vaccination, *Clostridioides difficile*, colonisation factor

## Abstract

*Clostridioides difficile* is the main cause of health-care-associated infectious diarrhoea. Toxins, TcdA and TcdB, secreted by this bacterium damage colonic epithelial cells and in severe cases this culminates in pseudomembranous colitis, toxic megacolon and death. Vaccines in human trials have focused exclusively on the parenteral administration of toxin-based formulations. These vaccines promote toxin-neutralising serum antibodies but fail to confer protection from infection in the gut. An effective route to immunise against gut pathogens and stimulate a protective mucosal antibody response (secretory immunoglobulin A, IgA) at the infection site is the oral route. Additionally, oral immunisation generates systemic antibodies (IgG). Using this route, two different antigens were tested in the hamster model: The colonisation factor CD0873 and a TcdB fragment. Animals immunised with CD0873 generated a significantly higher titre of sIgA in intestinal fluid and IgG in serum compared to naive animals, which significantly inhibited the adherence of *C. difficile* to Caco-2 cells. Following challenge with a hypervirulent isolate, the CD0873-immunised group showed a mean increase of 80% in time to experimental endpoint compared to naïve animals. Survival and body condition correlated with bacterial clearance and reduced pathology in the cecum. Our findings advocate CD0873 as a promising oral vaccine candidate against *C. difficile.*

## 1. Introduction

*Clostridioides difficile* is a Gram-positive, spore-forming anaerobic bacillus found in the intestinal tract of humans and certain animals (dogs, cats, pigs and birds) and in the environment. Approximately 5% of healthy adults and 15–70% of infants are colonised by *C. difficile* but its prevalence is much greater in hospitalised patients or nursing home residents [1]. The incidence and severity of *C. difficile* infection (CDI) has increased over the past 20 years [2] such that it is now the leading cause of hospital-acquired infection worldwide [3]. In the US, CDI accounts for 15% of all nosocomial infections [4]. The annual economic cost of CDI in the US is estimated to be $5.4 billion with $4.7 billion of the costs incurred in healthcare settings [5]. Risk factors associated with CDI are mainly antibiotic therapy [6] and age (over 65 years) [1], also immunosuppressive therapy, severe underlying illness, recent surgery and prolonged hospitalization or nursing home stay [7,8,9,10,11,12,13]. In addition, recent studies show that the incidence of community-acquired CDI is increasing [14].

The life cycle of *C. difficile* begins with the ingestion of spores from faecal–oral transmission. The spores are dormant and hardy and survive in the environment. When ingested, spores resist the proteolytic enzymes and acidity of the stomach and enter the intestine where they can persist [6]. Bile acids play an important role in inducing germination of these spores into metabolically active vegetative cells [15]. When the balance of microorganisms in the colon is disrupted due to antibiotics, the constitution of bile salts changes, which promotes germination. Furthermore, the colonisation resistance barrier is reduced giving *C. difficile* the opportunity to colonise the enterocytes and multiply [16]. *C. difficile* is a non-invasive pathogen and its virulence is attributed to enzymes such as collagenase and hyaluronidase and to its exotoxins, TcdA and TcdB. In the case of hypervirulent strains, a third toxin, *C. difficile* transferase (CDT or binary toxin) is produced. TcdA and TcdB are potent monoglucosyltransferases that damage the cytoskeleton of epithelial cells lining the colon, which leads to disruption of the tight junctions, fluid secretion and neutrophil infiltration [17].

The clinical presentation of CDI ranges from an asymptomatic carrier state to a mild or moderate condition (abdominal pain, fever, nausea and vomiting, weakness and loss of appetite) to life-threatening fulminant colitis. In severe cases, patients may present with pseudomembranous colitis, toxic megacolon, perforation of the colon, and septic shock [18]. The mortality rate directly attributed to CDI is approximately 5% but mortality associated with CDI complications reaches 15–25% and up to 34% in patients admitted to intensive care units [19].

Antibiotics are the mainstay of therapy [20]. Metronidazole has traditionally been the first-line drug for mild to moderate cases and vancomycin for severe and recurrent CDI [21]. As they are broad spectrum antibiotics, they dramatically reduce the amount of intestinal microbiota that play an important role in inhibiting germination of spores or in directly killing *C. difficile*. The resulting dysbiosis leaves patients vulnerable to a subsequent infection. A narrow-spectrum macrolide antibiotic, fidaxomicin, with comparable efficacy to vancomycin against *C. difficile* was approved in 2011. Fidaxomicin has no significant influence on the flora of the colon and is associated with a lower percentage of CDI recurrence than vancomycin. Fidaxomicin and vancomycin are now the cornerstone of CDI treatment [18,22]. Antibiotic treatments however have serious limitations. Firstly, they predispose patients to relapse particularly in the case of hypervirulent epidemic 027 strains. Recurrent CDI occurs in 10–25% of patients during the first week after completing treatment for the initial episode and increases up to 65% in patients who have already experienced relapse more than once [20,23]. Secondly, antibiotics impose selection pressure for the development of resistance. To minimise recurrence, faecal microbiota transplantation (FMT) may be offered to rapidly restore the gut flora. FMT along with withdrawal of antibiotics achieves the highest rate of prevention of recurrent CDI among all therapeutic options to date [24,25,26]. However, the potential for long-term side effects of FMT remain to be investigated.

In terms of natural protection, as high levels of antibodies against TcdA and TcdB have been shown to correlate with the prevention of primary CDI and low rates of recurrence [27,28], passive immunisation strategies were developed. Therapeutic benefits have been demonstrated for Bezlotoxumab, a monoclonal antibody specific for TcdB. However, the use of Bezlotoxumab is limited by high cost and its unavoidably transient action [29].

The only solution for long-term protection from primary CDI as well as recurrence is to actively immunise. Intra-muscular (i.m.) vaccines based on inactivated forms of TcdA and TcdB have been developed by Sanofi Pasteur and Pfizer, and on defined toxin fragments by Valneva. These formulations have proved safe and immunogenic, with high titres of toxin-neutralising serum antibodies detected. However, Sanofi Pasteur withdrew their vaccine from a phase III trial when data showed it failed to protect from CDI. Valneva has suspended their trial and results are pending for Pfizer’s phase III trial.

The majority of licensed vaccines for infectious diseases are administered parenterally either by subcutaneous or i.m. injection. The resulting immune response is generally limited to systemic humoral immunity with some cellular immunity, and only weak protection is generated at mucosal surfaces [30,31]. In contrast, vaccination at mucosal surfaces induces sIgA and cell-mediated immune responses, while still producing systemic IgG [32,33,34,35]. To immunise against gut pathogens, the most direct route to target the small intestine and generate local protective immune responses is the oral route, mimicking the natural infection route. Indeed, several oral vaccines have been licensed and have proven to be highly effective including those directed against typhoid, cholera, polio and rotavirus [31].

In order to investigate the oral route to immunise against *C. difficile*, a pilot study was conducted in hamsters. Enteric capsules were used to deliver two recombinant antigens to the small intestine. The hamster is a clinically relevant model for CDI as it manifests the full course of infection seen in humans. Moreover, being particularly susceptible to CDI, the hamster is a lethality model and thus an effective indicator of protective immunity [36]. One of the antigens chosen was the *C. difficile* colonisation factor CD0873. Following the initial identification of CD0873 as an immunoreactive protein in human infection [37], further studies in mice confirmed CD0873 to be an immunogenic surface-exposed lipoprotein that functions in adhesion of *C. difficile* to epithelial cells [38,39]. Another antigen (fragment) we chose was the terminal 514 amino acids of TcdB. Several studies have investigated the immunogenicity of individual portions of the two toxins given their large size. For both TcdA and TcdB, the region that is crucial for eliciting neutralising antibodies is the receptor binding domain (RBD) located at the C-terminus [40,41,42,43,44,45]. In short, the findings of our study advocate CD8730 as a promising vaccine candidate that when delivered orally stimulates a potent mucosal intestinal response with some protective efficacy afforded against CDI.

## 2. Materials and Methods

### 2.1. Bacterial Strains

*Escherichia coli* strains were purchased from New England Biolabs (NEB), Hitchin, UK; “NEB^®^ 5-alpha competent *E. coli*” was used for cloning purposes and “T7 Express competent *E. coli*” was used for recombinant protein expression. Strains were cultured aerobically in Luria Bertani (LB) broth with shaking or on LB agar (Fisher Bioreagents, Loughborough, UK) at 37 °C unless stated otherwise. Where appropriate, ampicillin was added to a final concentration of 100 µg/mL.

*Clostridioides difficile* strain 630 (PCR ribotype 012) was kindly provided by Peter Mullany, UCL. Strain R20291*ermB* was previously generated in which *ermB* conferring resistance to erythromycin was integrated into the genome of epidemic strain R20291 (PCR ribotype 027) [46]). Strains were cultured in Brain Heart Infusion medium (Oxoid) supplemented with 5 μg/mL yeast extract and 0.1% *w/v* L-cysteine (BHIS) containing selective supplements; 250 μg/mL D-cycloserine and 8 μg/mL cefoxitin (Oxoid) (BHIS CC). Strains were incubated overnight at 37 °C in an anaerobic workstation (Don Whitley Scientific, Bingley, UK) with an atmosphere of CO_2_ (10%), H_2_ (10%) and N_2_ (80%).

### 2.2. C. difficile Spore Preparation

BHIS broth was inoculated with a loop of colonies from an overnight BHIS CC agar culture and incubated anaerobically at 37 °C for 14–18 h. The culture was diluted 1:5 in BHIS broth and 100 µL were plated onto 5 BHIS plates. After a 5-day incubation, culture from all plates were scraped and re-suspended in 1 mL PBS. The suspension was heat shocked at 60 °C for 30 min to kill vegetative cells then centrifuged. The supernatant was discarded, and the pellet washed in 1 mL sterile H_2_O, centrifuged and the washes repeated 3 more times. The final pellet was re-suspended in 1 mL sterile H_2_O and stored at −80 °C. In order to quantify spores, 10^−1^ to 10^−8^ dilutions of stocks in PBS were plated onto BHIS CC containing 10 μg/mL sodium taurocholate and the vegetative cells that form colony forming units (CFU)s from germinated spores enumerated the next day.

### 2.3. Molecular Manipulations

Plasmid DNA was prepared using the Monarch^®^ Plasmid Miniprep Kit according to the manufacturer’s instructions (NEB). Enzymes for DNA manipulations included restriction endonucleases (NEB), T4 DNA ligase (NEB) and Calf Intestinal Alkaline Phosphatase (Invitrogen™, Thermo Fisher Scientific, Loughborough, UK). Gene cleaning was performed using the Monarch^®^ PCR & DNA Cleanup Kit (NEB) following the manufacturer’s instructions. DNA polymerase used for PCRs was Q5^®^ High-Fidelity DNA Polymerase (NEB) for cloning purposes or Taq DNA polymerase (NEB) for verification of constructs or for colony PCR. PCRs were performed in an Eppendorf^®^ Mastercycler^®^ (Stevenage, UK)). Reaction mixtures were typically subjected to initial denaturation at 94 °C for 5 min followed by 35 cycles: Denaturation at 94 °C for 30 s, annealing at the appropriate temperature for the primers for 30 s and extension at 72 °C for 30 s to 1 min depending on the length of amplicon, and a final extension cycle at 72 °C for 5 min. The amplified DNA was visualised by electrophoresis with 1% *w/v* agarose gels.

### 2.4. Engineering of Antigen Expression Constructs

The Intein Mediated Purification with an Affinity Chitin-binding Tag-Two Intein (IMPACT-TWIN) system was used (NEB), specifically the pTWIN1 vector, which was modified to incorporate a second affinity tag, 10x Histidine. Primers pET52bFor: 5′-GGTGGTGGATCCGCTGGTGCCACGCGGT-3′ and pET52bRev: 5′-GGTGGTGCTCAGCTTAGTGGTGGTGATGGTG-3′ were used to PCR-amplify the region incorporating the His tag from pET-52b (+) (Novagen, Merck Group, Feltham, UK) and the amplicon ligated into *Bam*HI-*Blp*I sites of pTWIN1. The construct was confirmed by sequencing using primers Ssp intein for: 5′-ACTGGGACTCCATCGTTTCT-3′ and His check seq Rev: 5′-ATAGTTCCTCCTTTCAGC-3′.

The nucleotide sequence of CD0873 and of the terminal fragment of the RBD of TcdB (AA 1852-2366) of *C. difficile* strain 630 were codon-optimised for *E. coli*. Additionally, the single cysteine found three-quarters of the way into the coding sequence of both antigens was substituted with alanine. This was to prevent any disulphide bond forming between this cysteine and the free cysteine at the N-terminus, which could impact on the tertiary structure of the recombinant protein potentially altering important immunological epitopes. The gene strings were chemically synthesised (Life Technologies, Thermo Fisher Scientific) and used as template for PCR with primers 0873 For: 5′-GGTGGTTGCTCTTCCAACTGTAGCCAAGGTGGTGATAG-3′ and 0873 Rev: 5′-GGTGGTTGCTCTTCCAACTGTAGCCAAGGTGGTGATAG-3′ and TcdB-RBDFor: 5′-GGTGGTTGCTCTTCCAACTGTATTACCGGT-3′ and TcdB-RBDRev: 5′-GGTGGTGGATCCTCGCTAATAACCAG-3′ respectively. The PCR products were digested with *Sap*I, *Pst*I or *Sap*I, *BamHI* and ligated to the *Sap*I-*Pst*I or *Sap*I-*BamHI* sites of pTWIN1-His and ligation mixtures used to transform NEB^®^ 5-alpha cells. Clones were confirmed by sequencing as above then transformed into T7 Express cells.

### 2.5. Expression and Purification of Recombinant Antigens by Immobilised Metal Affinity Chromatography (IMAC)

First, 1 L *E. coli* broth cultures *A*_600_ 0.6–0.7 were induced by adding isopropyl β-D-1-thiogalactopyranoside (IPTG) to a final concentration of 0.3 mM with shaking at room temperature overnight. Cells were harvested by centrifugation. The cell pellet was resuspended in ice-cold binding buffer (20 mM Tris-HCl, 1 M NaCl, 40 mM imidazole, pH 7.4), sonicated using the Fisherbrand™ Q500 Sonicator then centrifuged. The supernatant (cytosolic/soluble fraction) was harvested, and the pellet (insoluble fraction) re-suspended in binding buffer and the fractions analysed by sodium dodecyl sulphate polyacrylamide gel electrophoresis (SDS-PAGE) (2.9).

The soluble fraction was passed through a pre-charged Ni^2+^ PD-10 column (GE, Healthcare Life Sciences, Amersham, UK) containing Blue Sepharose 6 Fast Flow resin (GE, Healthcare). The flow-through was collected then the beads were washed with binding buffer to remove unbound proteins. The target protein was eluted with increasing concentrations of imidazole (50 mM, 100 mM, 250 mM and 500 mM) in elution buffer (0.1 M Tris-HCl and 2.5 M NaCl). All eluates were checked by SDS-PAGE (2.9) and those containing pure protein combined for dialysis in PBS at 4 °C overnight.

CDTcdB-RBD localised in the insoluble fraction and thus additional on-column solubilisation was required. The pellet was re-suspended in breaking buffer (binding buffer containing 500 mM NaCl and 6M guanidine HCl) then centrifuged. The supernatant was loaded onto Ni^2+^ columns equilibrated in breaking buffer then flushed sequentially with binding buffer containing 6 M urea, 4 M urea, 2 M urea, finally 1 mM reduced glutathione and 0.1 mM oxidised glutathione. The solubilised protein was then passed through a Ni^2+^ column and the protein purified as above.

All protein suspensions were standardised to 1 mg/mL in PBS and aliquoted in 1 mL volumes to which 1 mg of trehalose (cryoprotectant) was added as an excipient. Each aliquot was lyophilised by snap freezing in liquid nitrogen then freeze-dried overnight in a FreeZone^®^ Benchtop Freeze dryer 2.5 L (Labconco, Kansas City, MO, USA). Each powdered aliquot was packed separately into gelatin capsules, size 9 (Torpac Fairfield, NJ, USA) using the funnel, tamper and stand provided by the manufacturer.

### 2.6. Dissolution Assay to Optimise Enteric Coating

Optimisation of capsule coating was conducted by testing maximum resistance to dissolution in simulated gastric fluid, DILUT-IT™ (J.T.Baker, Avantor, Allentown, PA, USA) pH 1.2, and maximum susceptibility to dissolution in simulated intestinal fluid, PBS pH 6.7. Capsules were first packed with 22 mg of powdered Bromophenol Blue and glucose (Sigma, Merck Group, Feltham, UK) 1:2.5. The polymer solution described by Staelens et al., (2016) [47] was used; 12.5% EUDRAGIT L100 (Evonik Industries, Essen, Germany) in isopropanol with Triethyl citrate (TEC) (10% *w/v*) added as a plasticizer and the addition of 3% H_2_O (*w/w*) tested. One versus two coats were compared. In duplicate, capsules were individually placed in 5 mL of simulated gastric fluid and absorbance measurements taken over 5 h at 450 nm. The capsules were then transferred to 5 mL of simulated intestinal fluid and absorbance measurements were taken over 5 h at 590 nm.

### 2.7. Live CT Imaging to Track Location of Capsule Dissolution

Four capsules were packed with 25 mg BaSO_4_ and dip coated once in the optimised polymer solution: 12.5% EUDRAGIT L100 containing 10% TEC and 3% H_2_O. Four female Golden Syrian hamsters aged 12–16 weeks, weighing approximately 150 g were purchased from Janvier Labs (Le Genest-Saint-Isle, France), housed in individually vented cages (IVCs) then a week later given a single capsule orally via a Luer lock dosing applicator. For the acquisition of in vivo Computer Tomography (CT) scans, hamsters were anesthetised with 1.5% Isoflurane in 100% oxygen (AB) prior to each scanning time point; 1.5 h, 3 h and 5 h post administration then allowed to recover completely from each anaesthetic using O_2_. After the final recovery, animals were euthanised (2.16).

In vivo acquisition of CT scans was taken on a BioScan SPECT/CT and reconstructed on Nucline software (version 2.3, Mediso, Budapest, Hungary). Scans were acquired using the following scan parameters; 45 kVp-X-ray source voltage at 35 ms exposure time per projection resulting in a total scan time of 15 min. Reconstructed 3D 22 µm^3^ isotropic voxel data sets were generated, and images were processed using Vivoquant software (version 4.1, Invicro, a Konica Minolta Company, Boston, MA, USA).

### 2.8. In Vivo Immunogenicity Study

Female Golden Syrian hamsters aged 12–16 weeks, weighing approximately 150 g were purchased from Janvier Labs and housed in IVCs. Hamsters were randomly divided into 4 groups: Experimental group given capsules containing recombinant CD0873 in excipient (*n* = 4), experimental group given capsules containing recombinant CDTcdB-RBD in excipient (*n* = 4), non-immunised i.e., naïve negative control group (*n* = 2) and a group given capsules containing excipient only (*n* = 2). The purpose of this latter group was to check if the capsule or excipient contributed towards any immunogenicity detected in experimental groups. Oral dosing with capsules was performed on days 1, 15 and 30. Hamsters were euthanised 14 days post final immunisation (2.16). Blood was collected by cardiac puncture, left to clot overnight at 4 °C and serum harvested after centrifugation. A section of the ileum was fixed in 10% (*v/v*) neutral buffered formalin (NBF) (Sigma) for histological analysis (2.15). The remainder of the small intestine was placed in 5 mL PBS containing SIGMAFAST™ protease inhibitors (Sigma ), flushed through twice with 1 mL of this suspension then the supernatant collected after centrifugation. Serum and intestinal fluid were filter-sterilised and stored at −80 °C.

### 2.9. SDS-PAGE and Western Immunoblotting and Whole Cell Immuno-Dot Blotting

Procedures were performed as described previously [48] with 5% dry-milk (*w/v*) (Sigma) in Tris buffered saline containing 0.01% Tween (*v/v*) (TBST) for blocking. All antibodies were diluted in 1% *w/v* dry-milk in TBST and TBST used for washes. Samples (soluble and insoluble fractions of cell lysates, eluates, purified recombinant protein or intestinal fluid from hamsters) were added to 2X Laemmli sample buffer and fractionated by 10% (*w/v*) SDS-PAGE. Transfer to PVDF membranes was conducted using the Trans-Blot Turbo Transfer System (Bio-Rad, California, USA). Primary antibodies used were rabbit anti-His tag antibody (1:1000) from Cell Signaling Technologies, CST(Danvers, MA, USA) rabbit anti-CD0873 antibody [39] (1:5000) and mouse anti-TcdB antibody (1:1000) (The Native Antigen Company, Oxford, UK) to confirm antigens. Rabbit-anti-hamster secondary antibody was used to detect the heavy chain (H) of IgA (Brookwood Biomedical, Jemison, AL, USA). Briefly, IgA (H) antibody was purified from rabbit anti-hamster IgM, IgG, IgA (H) by cross absorption against hamster IgG and IgM (Brookwood Biomedical) and used at 1:1000 to detect vaccine-induced sIgA in intestinal fluid. Secondary antibodies used were anti-rabbit IgG horseradish peroxidase (HRP) (1:1000) (CST) and anti-mouse IgG HRP (1:1000) (CST). Protein bands were detected using 3,3′, 5,5′-Tetramethylbenzidine (TMB) substrate (Sigma) and visualised using the Gel Doc™ XR system (Bio-Rad, CA, USA). Immuno-dot blots were performed by spotting whole cell suspensions of *C*. *difficile* onto Nitrocellulose [48] then conducting the Western immunoblotting procedure described.

### 2.10. Indirect ELISA to Assess IgG Levels in Serum

Ninety-six--well Nunc MaxiSorp^™^ plates were coated with 100 µL purified recombinant proteins; CD0873 and CDTcdB-RBD at a concentration of 2.5 µg/mL in 0.2 M sodium bicarbonate, pH 9.4 and the proteins left to adsorb onto the wells overnight at 4 °C. All wash stages consisted of 5 washes with 200 µL PBS containing 0.01% Tween (PBST). Wells were first blocked with 200 µL of 5% dry-milk (Sigma) in PBST for 2 h at room temperature, washed then incubated over night at 4 °C after addition of 100 µL serum diluted 1:10 in PBST, in triplicate. Wells were washed then incubated for 2 h at room temperature in 100 µL goat anti-hamster IgG highly cross adsorbed-Biotin antibody (Sigma) diluted 1:20,000 in PBST, washed again then incubated for 2 h in Streptavidin-HRP (R&D Systems, Minneapolis, MN, USA) diluted 1:200 in PBST. *A*_650_ was measured after addition of 100 µL TMB substrate (Sigma) for 15 min using the CLARIOstar^Plus^ (BMG Labtech) Plate Reader.

### 2.11. Toxin Neutralization Assay

VERO cells were seeded at 1 × 10^4^ per well in a 96-well plate in 50 µL phenol red-free DMEM (Gibco™-ThermoFisher Scientific) supplemented with 4.5 g/L D-glucose, 584 mg/L L-glutamine, 25 mM HEPES, penicillin/streptomycin and 10% *v/v* fetal bovine serum (FBS) (Sigma) and incubated for 18–20 h at 37 °C in 5% CO_2_. Serum and intestinal fluid were serially diluted 2-fold (serum, 1:4 to 1:512 and intestinal fluid 1:128) in serum-free, phenol red-free DMEM and mixed with either an equal volume of TcdA at 200 ng/mL or TcdB at 1 ng/mL for 1 h at 37 °C. Neat and serial dilutions of serum or intestinal fluid-toxin mixtures were added to VERO cells in triplicates to give a total well volume of 100 µL and plates were incubated for 22 h at 37 °C. The final concentration of TcdA and TcdB was 50 ng/mL and 0.25 ng/mL respectively. Toxin neutralization was assessed by MTT assay. Briefly, all medium was removed from cells and 50 µL DMEM containing 0.5 mg/mL 3-(4,5-dimethylthiazol-2-yl)-2,5-diphenyltetrazolium bromide (MTT) was added to each well and incubated at 37 °C for 4 h. After removal of MTT solution, 75 µL DMSO was added to cells, and after 10 min the absorbance was read at 570 nm.

### 2.12. Adherence Blocking Assay

Caco-2 cells were cultured in Dulbecco’s Modified Eagles Medium (DMEM) (Gibco™-ThermoFisher Scientific) supplemented with 4.5 g/L D-glucose, 584 mg/L L-glutamine, 25 mM HEPES, 10% *v/v* FBS and penicillin/streptomycin in a humidified 5% CO_2_ atmosphere at 37 °C. Cells were seeded at 5 × 10^4^ cells per well in 24-well tissue culture plates (Corning). Monolayers were used 14 days after seeding with medium changed every 2–3 days. The culture medium was further changed 24 h prior to conducting the assay. The inoculum was prepared by standardising the optical density (OD) of a 10 mL overnight broth culture of *C. difficile* strain 630 to *A*_600_ 0.6, centrifuging and washing the cells with PBS and re-suspending the pellet in non-supplemented DMEM. Caco-2 monolayers were infected at a multiplicity of infection (MOI) of 1:5 and 1:20 in triplicate. To confirm the MOIs, 10^−1^ to 10^−7^ dilutions of the cell suspension in PBS were plated on BHIS CC for enumeration of CFUs. The adherence assay was performed under anaerobic conditions at 37 °C. 50 µL of serum or intestinal fluid diluted 1:5 and 1:2 respectively, in non-supplemented DMEM were added to 50 µL of the bacterial cell suspension and incubated for 1 h. This mixture was then added to Caco-2 cells in triplicate following removal of the medium in the wells. After 2 h of incubation, non-adherent bacteria were removed by pipetting and adherent bacteria harvested as follows. Caco-2 cells were washed 3 times with PBS, incubated in 200 µL 1X trypsin-EDTA to detach them from the wells then re-suspended in 300 µL supplemented DMEM. 10^−1^ to 10^−3^ dilutions of cells in PBS were plated on BHIS CC plates and CFU enumerated the following day.

### 2.13. In Vivo Challenge Study

Female Golden Syrian hamsters aged 12–16 weeks, weighing approximately 150 g, were purchased from Janvier Labs and housed in IVCs. Hamsters were randomly divided into 3 groups: Naïve negative control group (*n* = 2), group immunised orally with capsules containing recombinant CD0873 in excipient (*n* = 4) and a group given toxoids (mock Sanofi Pasteur vaccine) (*n* = 2). The toxoid vaccine consisted of formalin-inactivated TcdA and formalin-inactivated TcdB (Native Antigen Company, Oxford, UK) at 5 µg/dose resuspended in an alum adjuvant in PBS [49]. This group was given an i.m. injection after anaesthesia with Isoflurane (AB) on the same days as the experimental group that was immunised orally.

Two weeks after the final immunisation (2.8), hamsters were orally gavaged with 100 µL clindamycin (30 mg/kg) to disrupt their gut microbiota then challenged 5 days later with 10^3^ spores of strain R20291*ermB* in 100 µL of PBS. Animals were monitored several times daily for symptoms of CDI. In addition to monitoring weight (with a loss of 20% between consecutive checks or 10% decrease at 2 consecutive checks resulting in euthanasia), other parameters scored were wet tail or loose faeces, hunched posture, piloerection and reduced activity and reduced response to stimuli. Scores of 0–3 were given for each parameter (0 being no change and 3 being a major change) and animals euthanised once CDI symptoms reached a threshold score of 15 [50] or once the experimental endpoint was reached (2 weeks post infection). The bacterial load was monitored throughout the study by enumerating spores shed in faeces (2.14) and the histopathology of the cecum scored at the endpoint (2.15).

### 2.14. Monitoring the Burden of C. difficile

Bacterial burden over time was monitored by enumerating the number of spores shed in faeces, which are released by sporulating vegetative cells (one spore per vegetative cell). Unlike vegetative cells that die upon exposure to O_2_ in the air, spores within faeces survive. By plating faecal homogenates on BHIS CC containing sodium taurocholate (2.2), the vegetative cells that form CFUs from germinated spores can be enumerated the next day. One millilitre of PBS was added to 100 mg of faecal pellet in a 2 mL Precellys^®^ CK28 tube and homogenised using the Precellys^®^ 24 Tissue Homogenizer (Bertin Instruments, Montigny-le-Bretonneux, France). Suspensions were then centrifuged and the supernatant containing spores harvested and stored at −80 °C. Serial dilutions were spotted in triplicate onto plates as described above and CFU enumerated. Confirmation of colonisation with the infecting strain, R20291*ermB*, was obtained by colony PCR with primers Cdi-630-pyrD-sF1 and ermB-HindII-R and the sequence of amplicons verified using the same primers [46].

### 2.15. Histopathological Assessment of Cecum

Intestinal sections were fixed in 10% NBF, processed overnight on a 14 h programme in a TP1020 Automatic Benchtop Tissue Processor (Leica Biosystems, Newcastle, UK), paraffin embedded in a Histostar Embedding Workstation (Thermo Fisher Scientific) and sectioned at 5 µm, mounted on glass slides and Hematoxylin and Eosin (H&E) stained (Leica ST 5020). Blinded analysis was conducted by an experienced pathologist using an established scoring system [51]. Sections were assessed for oedema (0–3), neutrophil infiltration (0–3) and epithelial tissue damage (0–3) with 0, normal and 3, severe.

### 2.16. Ethics Statement

Animal studies were devised usingthe Experimental Design Assistant (EDA) online tool [52] and conducted in strict accordance with the requirements of the Animals Scientific Procedure Act 1986. Prior approval for these procedures was granted by the University of Nottingham Animal Welfare and Ethical Review Body and by the UK Home Office under project license PPL P4712E8BB. Animals were euthanised by CO_2_ inhalation followed by cervical dislocation to minimize suffering.

### 2.17. Statistical Analysis

The non-normally distributed data from this study were analysed as follows. For the immunogenicity data: Antibody titre and adherence blocking, a non-parametric Mann–Whitney U test was conducted. For clinical outcome following immunisation and challenge, data for time to endpoint, weight, bacterial load and histopathology were analysed by a non-parametric Mann–Whitney U test. For animal survival data, a Kaplan–Meier survival analysis was conducted using a log-rank (Mantel–Cox) test. All statistical tests were performed using GraphPad version 7 (San Diego, CA, USA) and *p* values of less than 0.05 were considered to indicate statistical significance.

## 3. Results

### 3.1. Purification of RecombInant Protein Antigens with an N-terminal Cysteine by Novel Double Affinity Tagging

The colonisation factor CD0873 is a conserved, surface-exposed lipoprotein [39]. Lipoprotein precursors harbour an N-terminal signal peptide ending in a lipobox with a terminal cysteine. Following lipidation of this cysteine, cleavage of the signal peptide occurs at the residue immediately before this such that the acylated cysteine forms the new N-terminus. The signal peptide of CD0873 (strain 630) is MINKKRLASLILAGALSISMLTGC as predicted by lipoprotein prediction programmes (LipoP 1.0, SignalP-5.0 and DOLOP).

In order to obtain the cleaved version of CD0873, the form that would be expressed on vegetative cells, the IMPACT-TWIN system was used as it enables purification of recombinant proteins possessing an N-terminal cysteine. This is achieved by N-terminal fusion of the target protein to an intein bearing a chitin-binding domain (CBD). The inducible self-cleavage activity of the intein separates the target protein from the affinity tag. For consistency, we used the same system to express and purify the terminal fragment of TcdB.

**Figure 1:**
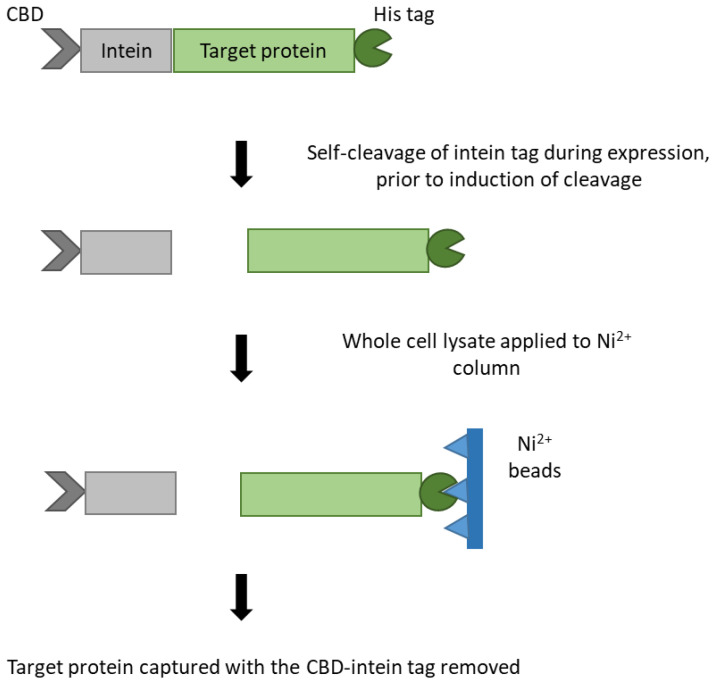
Schematic diagram of pTWIN1-His expression system. The expression vector pTWIN1 (NEB) was modified by the inclusion of a Hig tag for C-terminal tagging. This prevented loss of the target protein due to excessive natural self-cleavage of the N-terminal intein tag bearing the chitin-binding domain (CBD) by trapping the target protein with a Ni^2+^ column by Immobilised Metal Affinity Chromatography (IMAC).

However, due to excessive self-cleavage without induction, prior to purification, the target protein could not be trapped by chitin beads and was lost in the flow-through. By incorporating a His tag in the pTWIN1 vector for C-terminal tagging, Ni^2+^ columns could be used instead of chitin columns to trap the recombinant protein now bearing the N-terminal cysteine (Figure 1).

Briefly, the region incorporating the thrombin cleavage site and the His tag in pET52b (+) was cloned into pTWIN1 to create pTWIN1-His and this new vector was used to produce recombinant CD0873 and CDTcdB-RBD. The nucleotide sequence of both antigens was taken from *C. difficile* strain 630 (NCBI), codon-optimised for *E. coli* and chemically synthesised before cloning into pTWIN1-His.

**Figure 2:**
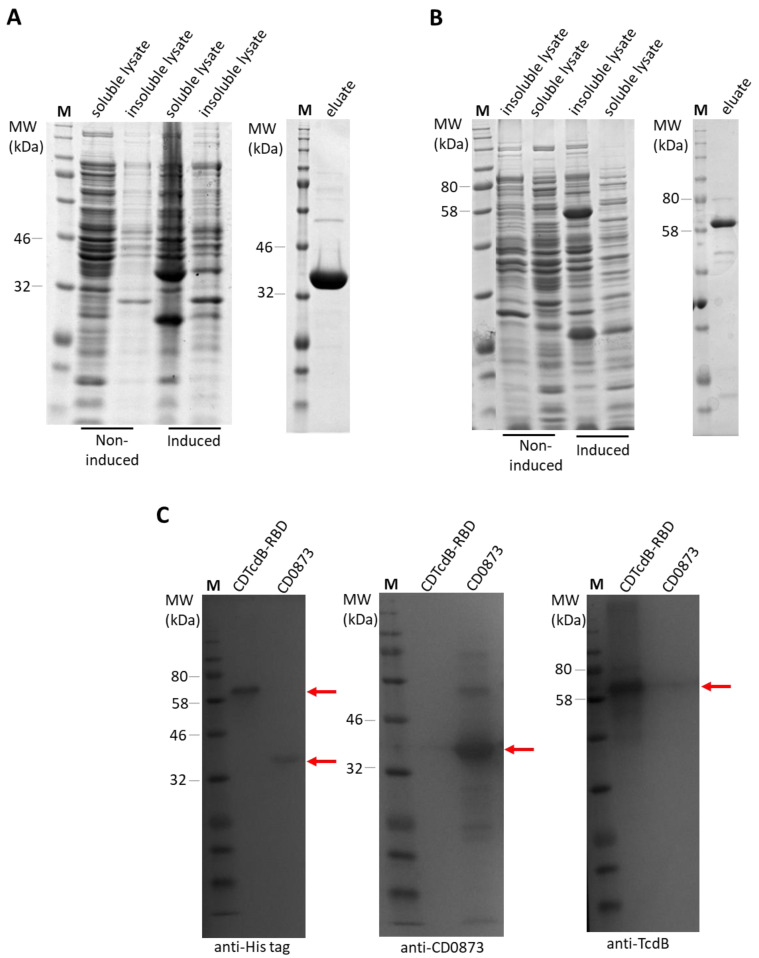
Purification of recombinant protein antigens. Protein extracts of soluble and insoluble fractions from induced and non-induced cultures of recombinant *E. coli* strain T7 and the eluates obtained after IMAC were separated by SDS-PAGE (10% acrylamide) and stained by Coomassie Blue (**A**,**B**) or analysed by Western immunoblotting (**C**). (**A**) Identification of CD0873 in the soluble fraction, (**B**) Identification of CDTcdB-RBD in the insoluble fraction, (**C**) Western immunoblot of purified recombinant CD0873 and CDTcdB-RBD probed with anti-His tag antibody, anti-CD0873 antibody or anti-TcdB antibody and detected by the addition of the appropriate HRP-conjugated secondary antibody and TMB substrate. Lane M: Colour pre-stained protein standard. Bands of expected molecular weight of 36 kDa for CD0873 and 61 kDa for CDTcdB-RBD were detected as indicated by the red arrows.

Following induction of protein expression and affinity purification, SDS-PAGE revealed a single band of expected molecular weight for each antigen; 36 kDa for CD0873 and 61 kDa for CDTcdB-RBD (Figure 2A,B). Confirmation of the proteins was obtained by Western immunoblotting, probing with anti-His tag antibody, anti-CD0873 antibody or anti-TcdB antibody and the bands detected with HRP-conjugated secondary antibodies and TMB substrate (Figure 2C). Final dialysed protein suspensions were diluted to 1 mg/mL in PBS, aliquoted into 1 mL volumes and cryoprotectant trehalose added as an excipient. Each aliquot was lyophilised then packed into separate Torpac gelatin capsules and stored at room temperature until required.

### 3.2. Single Dip Coating of Gelatin Capsules with EUDRAGIT L100 Mixed with TEC and Water Is Optimal for Targeted Release within the Small Intestine

**Figure 3:**
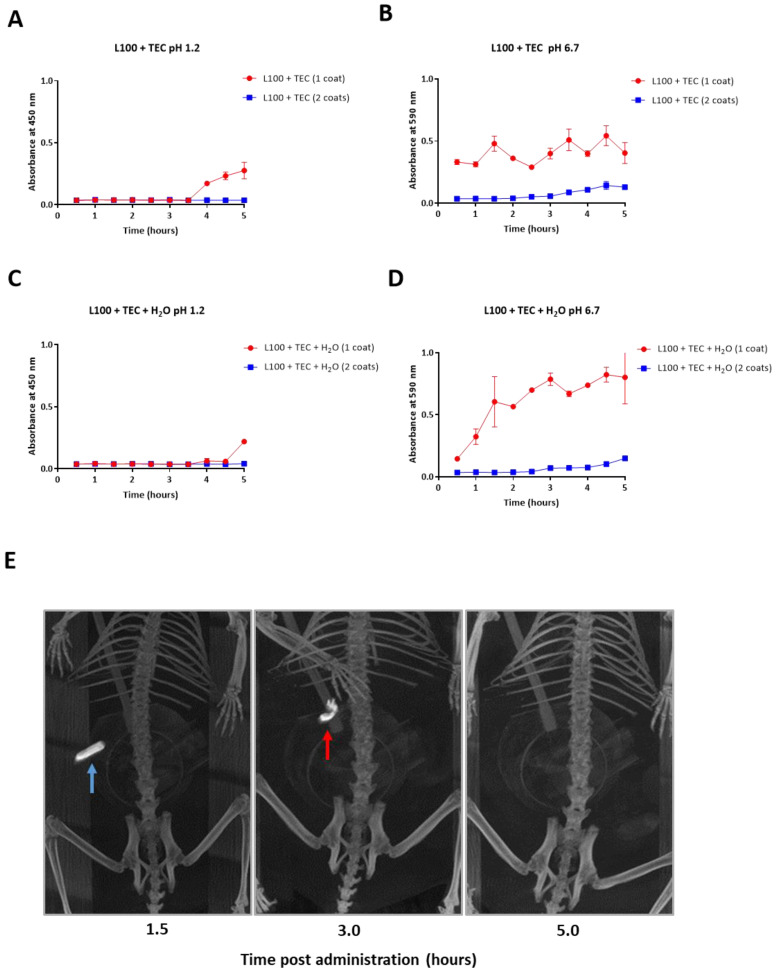
Dissolution of coated capsules in different pH conditions in vitro (**A**–**D**) and in vivo (**E**) to establish the optimal enteric coating required for targeted release of formulation in the small intestine. An in vitro assay was conducted to compare enteric capsules dip coated once or twice in EUDRAGIT^®^ L100 in 10% TEC without the addition of 3% H_2_0 (**A**,**B**) and with 3% H_2_0 (**C** and **D**). Dissolution of the capsules was tested in simulated gastric fluid pH 1.2 over 5 h (**A**,**C**) then in stimulated intestinal fluid pH 6.7 over 5 h (**B**,**D**) by measuring the changing absorbance of surrounding solutions over time due to release of Bromophenol Blue from the capsules. Error bars represent standard error of the mean (SEM). (**E**) Representative CT images in one hamster out of 4, showing localisation and dissolution of optimally coated capsule containing BaSO_4_ over time after oral administration. The capsule is found intact at the level of the stomach at 1.5 h (blue arrow) indicating stability of the coated capsule at the low stomach pH. At 3 h the capsule is entering the small intestine (red arrow) and is disintegrating at the higher intestinal pH. At 5 h the capsule has completely disintegrated, and its contents released and fully solubilised.

In order to establish the most appropriate enteric coating to resist dissolution in the acidic environment of the stomach but allow dissolution at the higher pH of the intestine for targeted delivery of the encapsulated protein to the small intestine, 12.5% (*w/v*) EUDRAGIT L100 was dissolved in isopropanol and mixed with 10% TEC [47]. The addition of 3% H_2_O (*v/v*) to enhance the plasticity of the polymer was tested. Capsules were first packed with powdered Bromophenol Blue and glucose then dip-coated either once or twice for comparison. Capsule dissolution was monitored over 5 h in simulated gastric fluid, pH 1.2 then over 5 h in simulated intestinal fluid, pH 6.7 (Figure 3A–D). Capsules dip coated once in L100 mixed with 10% TEC and 3% H_2_O resisted dissolution at pH 1.2 the greatest (Figure 3C) and subsequently dissolved the fastest at pH 6.7 (Figure 3D). For all subsequent in vivo studies, capsules were dip-coated once in this formulation.

To investigate targeted delivery to the small intestine in vivo, capsules were packed with BaS0_4,_ dip-coated once in the optimised polymer mixture then a single capsule was given orally to hamsters (*n* = 4). CT of the same capsule and animal was performed to track localisation of the capsule over time. The BaS0_4_-containing capsule was clearly visible as an intact capsule in the stomach 1.5 h after administration (Figure 3E). Partial degradation of the capsule and release of mainly non-dissolved BaS0_4_ in the small intestine was observed at 3 h. The capsule had completely disintegrated and disappeared at 5 h and the BaS0_4_ dispersed and fully solubilised in the intestinal fluid (Figure 3E). The in vivo findings corroborate our in vitro results that the capsule coated with L100 mixed with 10% *w/v* TEC and 3% H_2_O resisted the low pH of the stomach and disintegrated at the higher pH of the small intestine.

### 3.3. Mucosal and Systemic Antibody Responses Are Detected in CD0873-Immunised Hamsters

The hamster model is widely recognised as a stringent and relevant model for the evaluation of novel therapies and vaccines against *C. difficile* [49]. Hamsters were dosed orally, one capsule per dose, on days 1, 15, and 30 then euthanised two weeks later. One experimental group was immunised with capsules containing 1 mg of lyophilised CD0873 in trehlaose and another group with capsules containing 1 mg of lyophilised CDTcdB-RBD in trehlaose. Negative control groups included naive hamsters or hamsters given capsule containing excipient, trehalose only. At the experimental endpoint, a section of the ileum (the mucosal inductive site) was taken for histological analysis, and serum and intestinal fluid were harvested. Histological analysis revealed no inflammation of the ileum in any of the hamsters indicating good safety of all formulations).

**Figure 4:**
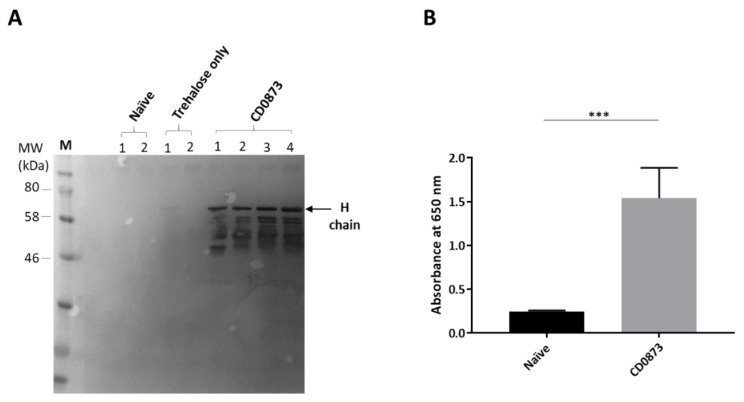
Immune responses in hamsters immunised orally with CD0873. Intestinal fluid and serum were harvested from hamsters vaccinated with recombinant CD0873 and from hamsters given capsule containing excipient only as well as from naïve hamsters and tested for the presence of sIgA and IgG, respectively. Intestinal fluid was extracted by flushing the small intestine with 5 mL of PBS containing protease inhibitors and serum was taken by cardiac puncture at the experimental endpoint. Intestinal fluid was further diluted 1:2 in sample buffer and serum was diluted 1:10. (**A**) Intestinal fluid (5 µL) from each hamster was fractionated by SDS-PAGE along with Colour Prestained Protein standard (NEB) and transferred to a PVDF membrane. The membrane was probed with rabbit anti-hamster IgA antibody (1:1000) and bands detected with anti-rabbit IgG HRP (1:1000) and TMB substrate. (**B**) Serum was pooled separately for the CD0873-vaccinated group and naïve group and tested for IgG by indirect ELISA. Goat anti-hamster IgG highly cross adsorbed-Biotin antibody (1:20,000) and Streptavidin-HRP (1:200) were used for detection. Data were obtained from two independent experiments, each with three technical replicates. Error bars represent standard error of the mean (SEM). A non-parametric Mann-Whitney U test was performed to compare groups. Statistical difference *p* value; (***) *p* < 0.001.

To investigate the prevalence of sIgA in the small intestine, diluted intestinal fluid of each hamster was probed by Western immunoblotting with rabbit-anti-hamster IgA (H) antibody and detected by anti-rabbit IgG HRP. A strong immuno-reactive band was observed for each of the CD0873-immunised hamsters corresponding to the molecular weight expected for the H chain of hamster IgA, reported previously to run between 51 kDa and 86 kDa [53] (Figure 4A). Given the very little fluid that can be extracted from the thin tubing of the small intestine of hamsters, a considerable volume of PBS (5 mL) was used to flush out the antibodies and this was further diluted 1:2 in sample buffer for fractionation. Despite being heavily diluted, the intense band observed for each immunised animal corresponding to the size of the H chain suggests a high titre of sIgA in the small intestine. No band was detected for any of the negative control group animals (Figure 4A). Likewise, there was no detection of sIgA in intestinal fluid of CDTcdB-RBD-immunised animals). As with naive animals, since no background level of antibody was detected for animals given capsules containing excipient only, this group was excluded from further assays.

**Figure A1:**
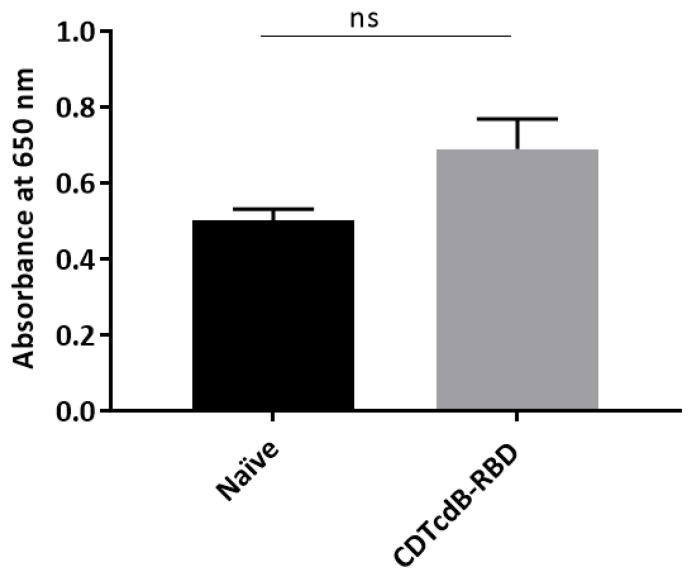
Detection of IgG in serum by indirect ELISA from hamsters immunised with CDTcdB-RBD and from naïve animals. Serum was pooled separately for the two groups and used at a 1:10 dilution. Goat anti-hamster IgG (H + L) highly cross adsorbed-Biotin antibody (1:20,000) and Streptavidin-HRP (1:200) were used for detection. Data were obtained from 2 independent experiments, each with 3 technical replicates. Error bars represent standard error of the mean (SEM). A non-parametric Mann-Whitney U test was performed to compare groups and non- significance (ns) found (*p* > 0.05).

In order to test whether immunised hamsters further generated a systemic immune response, pooled sera from immunised or naive hamsters were compared by indirect ELISA. Wells were first coated in recombinant CD0873 or CDTcdB-RBD then incubated in diluted serum. Biotin-labelled goat anti-hamster IgG was added and Streptavidin-HRP used for detection. A significantly higher titre of CD0873-specific IgG was detected in CD0873-immunised sera compared to naive animals *p* = 0.0001 (Figure 4B). Conversely, a non-significant difference in CDTcdB-RBD-specific IgG titre was observed between CDTcdB-RBD immunised and naive sera, *p* = 0.1797 (Figure A1). Toxin neutralisation assays with Vero cells were performed with sera from CDTcdB-RBD-immunised hamsters, but no neutralisation was observed. We conclude that only recombinant CD0873 was immunogenic and successfully stimulated both mucosal and systemic antibody responses.

### 3.4. Intestinal Fluid and Serum of CD0873-Immunised Hamsters Inhibits the Adherence of C. difficile to Caco-2 Cells

**Figure 5:**
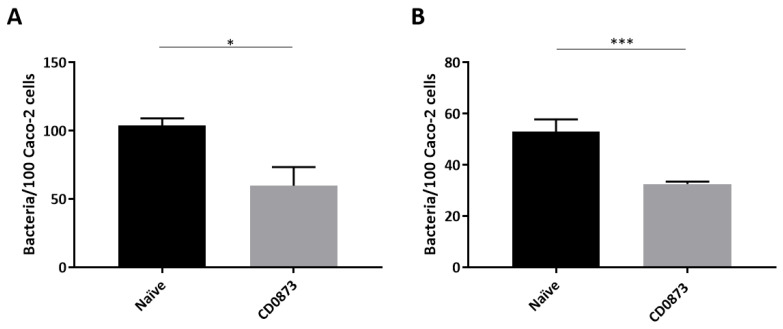
Adherence of *Clostridioides difficile* to Caco-2 cells is reduced by CD0873 antibodies. Adherence of *C. difficile* to Caco-2 monolayers after the bacterial cells were pre-incubated with (**A**) intestinal fluid diluted 1:2 and (**B**) sera diluted 1:5 from CD0873 orally immunised hamsters or from naïve hamsters. Cell-binding was measured by enumerating CFU from washed Caco-2 cells. The values are mean numbers of adherent bacteria per 100 Caco-2 cells. The assay was performed in triplicate. Error bars represent standard error of the mean (SEM). A non-parametric Mann–Whitney U test was performed to compare groups. Statistical difference *p*-value; (*) *p* < 0.05 and (***) *p* < 0.001.

To test if CD0873-induced antibodies in the intestinal fluid and in serum function in inhibiting colonisation of *C. difficile* to epithelial cells, Caco-2 cells were infected with *C. difficile* strain 630 pre-incubated with pooled intestinal fluid diluted 1:2 or pooled sera diluted 1:5. Two hours post infection, cells were washed and detached from wells with Trypsin then serial dilutions plated and CFUs enumerated. There was a significant reduction in the number of adhered cells of *C. difficile* when pre-incubated with intestinal fluid from immunised animals compared to naive animals, *p* = 0.0320 (Figure 5A). An even greater reduction in adherence was observed when *C. difficile* was pre-incubated with sera from immunised animals, *p* = 0.0001 (Figure 5B). Our findings suggest that CD0873-induced antibodies generated locally in the gut and systemically function in reducing the colonisation of *C. difficile* to epithelial cells.

### 3.5. Hamsters Immunised Orally with CD0873 Are Partially Protected from C. difficile Infection

**Figure A2:**
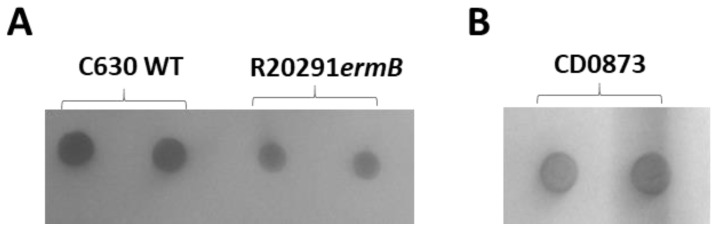
Immuno-dot blot of whole cells of *Clostridioides difficile* probed with rabbit anti-CD0873 antibody [39] to confirm surface expression of CD0873 on the hypervirulent strain R20291*ermB* used for challenge studies. (**A**) Strain 630, with known CD0873 surface expression [39], was used as a positive control for comparison to strain R20291*ermB*. Cells from overnight plate cultures were resuspended in 100 µL PBS and 5 µL spotted onto Nitrocellulose in duplicate. (**B**) Recombinant CD0873 was used as a positive control with 5 µL (2 ng/µL)) spotted in duplicate. The membranes were blocked in 5% dried milk in TBST for 1 h then incubated with rabbit anti-CD08730 antibody (1:5000) then in anti-rabbit IgG HRP (1:1000), with both antibodies diluted in 1% dried milk in TBST. Spots were detected by TMB substrate and visualised using the Gel Doc™ XR+ system with the Image Lab software (Bio-Rad).

Hamsters were immunised orally as before with CD0873 and compared to the naïve group. Another group was given formalin-inactivated toxoids i.m. (mock Sanofi Pasteur vaccine) for comparison [49]. Hamsters were given clindamycin by oral gavage to disturb their gut microbiota then infected five days later by oral gavage with 10^3^ spores of a hypervirulent strain of *C. difficile*. Specifically, R20291*ermB* was used since *ermB* confers a degree of resistance to clindamycin (in addition to erythromycin) thus avoiding killing of the inoculum by residual antibiotic at the time of infection [50]. Another benefit of using R20291*ermB* is to facilitate confirmation of the infecting strain by PCR detection of *ermB* in fecal homogenates. Surface expression of CD0873 on cells of strain R20291*ermB* was verified by whole cell immuno-dot blotting with anti-CD0873 antibody [39] prior to use in the hamster model (Figure A2). After infection, animals were monitored frequently for signs of CDI. Changes in body condition were scored and animals euthanised once a threshold score was reached as a result of fulminant CDI [54]. The experimental endpoint was two weeks post infection.

**Figure 6:**
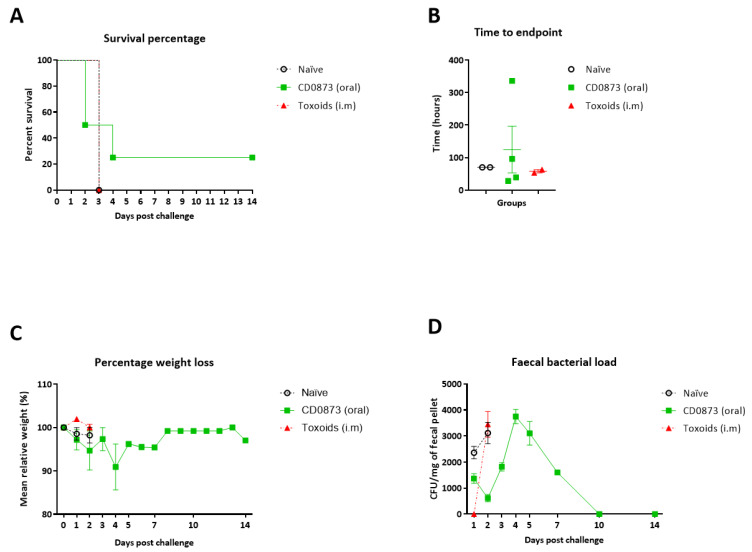
Clinical outcome of hamsters following oral vaccination with recombinant CD0873 and challenge with a hypervirulent strain of *Clostridioides difficile***.** Dosing was conducted on days 1, 15, and 30 orally for CD0873 vaccinated group and i.m. for the toxoid group and outcomes compared with the naïve group. Hamsters were given clindamycin (30 mg/kg) orally to disturb their gut microbiota two weeks after the third immunisation then challenged orally five days later with 10^3^ spores of R20291*ermB*. (**A**) survival percentage of animals, (**B**) time to end point of each animal, (**C**) percentage weight loss from starting weight and (**D**) counts of *C. difficile* spores shed in faeces post challenge. Error bars represent standard error of the mean (SEM). Data for the CD0873 vaccinated group was compared with the naïve group to investigate significance. For A, a Kaplan–Meier survival analysis was conducted using a log-rank (Mantel-Cox) test. For B, C and D, a non-parametric Mann–Whitney U test was used. Non-significance (*p* > 0.05) was reported for all data sets.

**Figure A3:**
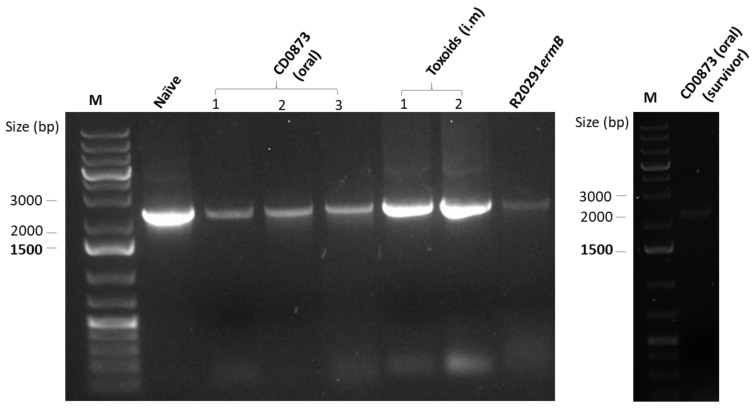
PCR confirmation of *Clostridioides difficile* R20291*ermB* strain from challenged hamsters. Agarose gel electrophoresis of PCR products obtained using primers specific for *ermB*; Cdi-630-pyrD-sF1 and ermB-HindII-R to amplify *ermB* of R20291*ermB* [46] directly from faecal homogenates as a diagnostic test for the presence of the strain used for infection. The amplicon obtained was the size expected [46] and was confirmed by DNA sequencing using the same primers.

The mean time to endpoint for the CD0873 orally immunised group was 80% greater than the naive group. One of the four CD0873-vaccinated hamsters displayed no symptoms of infection (Figure 6A,B). This hamster showed a normal, non-enlarged cecum post-mortem. Another of the CD0873-vaccinated animals showed only mild symptoms but was euthanised 96 h post challenge (Figure 6A,B). Post-mortem confirmed only minor swelling of the cecum. All other animals succumbed to disease within 70 h of being challenged and demonstrated highly enlarged ceca and loose faeces (Figure 6A,B). All hamsters progressively lost weight up to the endpoint except for the CD0873-vaccinated hamster that did not develop any clinical symptoms (survivor) (Figure 6C). After initially losing up to 10% of its initial weight over four days post challenge, it regained its weight within the next four days (Figure 6C). Its weight profile inversely correlated with bacterial load with CFU counts peaking at four days post challenge and diminishing after this with no shedding observed beyond day 10 (Figure 6D). Hamsters immunised i.m. with toxoids showed no protection.Confirmation of the presence of the infecting strain in all challenged animals was acquired by PCR detection of *ermB* from faecal homogenates (Figure A3) and verified by sequencing.

Although non-significance was determined for the vaccinated group, a trend in protection was clearly evident. The non-significance observed is likely reflective of the lack of statistical power due to the small *n* values used. To investigate the condition of the cecum of the fully protected hamster compared to that of naïve hamsters or hamsters given toxoids that succumbed to infection, the ceca were compared for histopathological differences.

### 3.6. Histological Examination of the Cecum Shows Reduced Pathology in CD0873-Immunised Hamsters

**Figure 7:**
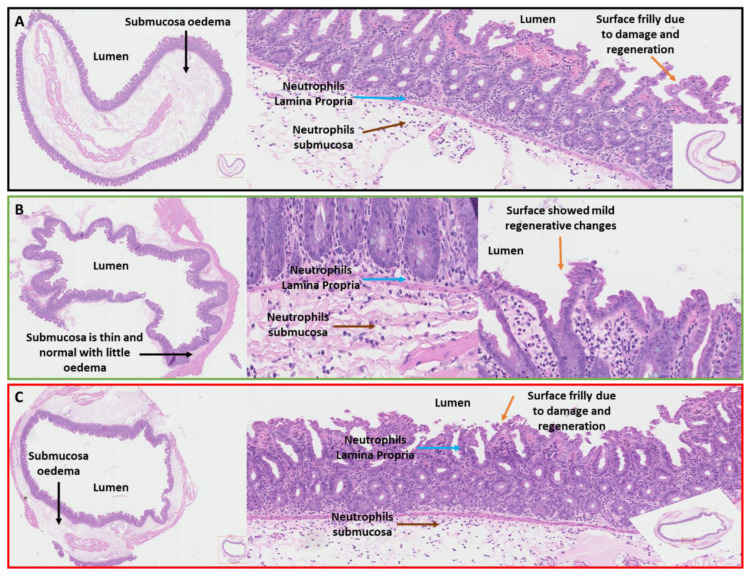
Histopathology of ceca following oral vaccination of hamsters with recombinant CD0873 and challenge with a hypervirulent strain of *Clostridioides difficile*. Cecum sections were taken from naïve hamsters, hamsters vaccinated orally with recombinant CD0873 and hamsters vaccinated i.m. with toxoids and H&E stained. Images of the CD0873-vaccinated hamster that displayed no clinical symptoms of CDI are shown (**B**) and compared with representative images for the naïve group (**A**) and toxoids group (**C**). Blue arrows indicate neutrophils in the Lamina Propria and brown arrows, neutrophils in the submucosa. Black arrows show oedema and orange arrows, the epithelium. In the CD0873-vaccinated hamster, there is far less neutrophil infiltration than seen in the toxoids group or naïve group. Likewise, very little oedema is observed for the CD0873-vaccinated hamster and the epithelium appears relatively intact in contrast to the toxoids vaccinated and naïve groups, which showed extensive oedema and damaged epithelium.

**Figure A4:**
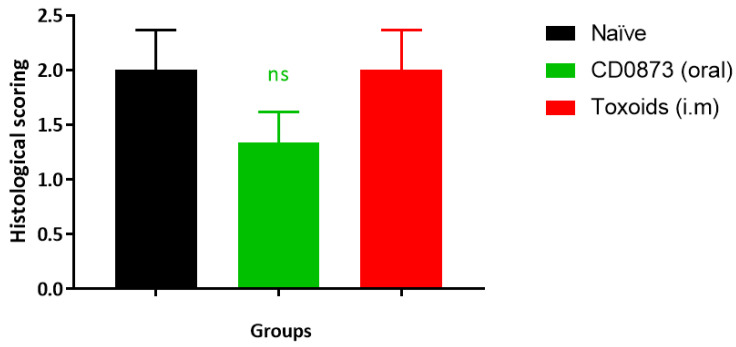
Combined histopathological scores for ceca following oral vaccination of hamsters with recombinant CD0873 and challenge with a hypervirulent strain of *Clostridioides difficile*. Sections were taken from naïve hamsters, hamsters vaccinated orally with recombinant CD0873 and hamsters vaccinated i.m. with toxoids. Scoring included oedema (0–3), neutrophil infiltration (0–3) and tissue damage (0–3) for each animal group with 0, normal and 3, severe. Combined scores of the 3 groups were compared by a non-parametric Mann–Whitney U test and non significance (ns) detected (*p* > 0.05).

For each hamster, a small portion of the cecum was taken, fixed, embedded and sectioned then mounted on slides and H&E stained. Sections were assessed for oedema (0–3), neutrophil infiltration (0–3) and tissue damage (0–3) with 0, normal and 3, severe. Scores for the 3 parameters were combined for each group. As expected non-significance was observed (Figure A4), however the CD0873-immunised hamster that was protected from CDI showed profoundly reduced pathology compared to naïve animals or animals given toxoids. The submucosa layer remained thin, the epithelium was relatively preserved and there were markedly fewer neutrophils (Figure 7B). Conversely, the toxoids-vaccinated and naïve animals both showed extensive oedema, damaged epithelium and an abundance of neutrophils in the Lamina Propria and submucosa (Figure 7A,C).

## 4. Discussion

Natural protection against extracellular mucosal pathogens requires primarily the production of sIgA antibodies that prevent attachment of pathogens to the mucosa and can furthermore neutralise the exotoxins [55,56]. Large complexes form of pathogens bound by sIgA, which get entrapped by mucous and cleared. A secondary systemic response often follows whereby serum IgG antibodies control mucosally invasive and systemically invasive pathogens by directly activating complement-mediated killing and by opsonophagocytosis [57].

Early studies investigating natural immune responses to CDI focused on testing sera for these secondary systemic responses. It was found that high titres of TcdB-specific serum IgG correlated with recovery from CDI without relapse and similar observations were reported for TcdA antibodies [58,59]. Conversely the inability to mount serum anti-toxin antibodies was associated with increased risk of recurrent CDI [58,60]. Many preclinical studies ensued whereby hamsters and mice were parenterally immunised with toxin-based formulations to induce toxin-neutralising serum IgG antibodies and different degrees of protection were reported [42,44,49,61,62,63,64,65,66]. However, results from human trials administering toxoid formulations parenterally have not supported the protective efficacies observed in preclinical studies, with Sanofi Pasteur terminating their phase III trial in 2017.

Oral immunisation on the other hand is a far more effective route for directly stimulating intestinal sIgA responses [67]. Licensed oral vaccines against polio, rotavirus, *Salmonella typhimurium* and *Vibrio cholera* 01 consistently demonstrate sIgA, as well as serum antibody and cellular immune responses, with strong protection against mucosal infection [68].

To this end, a pilot study in hamsters was conducted to test two recombinant antigens: Colonisation factor CD0873 and the terminal portion of TcdB, CDTcdB-RBD. Oral immunisation with CD0873 resulted in a high titre of sIgA in intestinal fluid and IgG in serum compared to naive animals, which significantly reduced the adherence of *C. difficile* to Caco-2 cells. With evidence of a mucosal antibody response, as well as a systemic humoral response against CD0873, we tested the protective efficacy of this antigen delivered orally. For the challenge strain, we chose heterologous R20291*ermB* derived from hypervirulent isolate R20291 () that is associated with severe disease, responsible for two outbreaks of CDI in the UK [69].

The orally immunised CD0873 group was partially protected with a mean increase of 80% in time to endpoint compared to the naive group (Figure 6A,B). One of the vaccinated hamsters was fully protected from CDI and displayed no symptoms other than up to 10% loss in weight, which was regained (Figure 6C). The weight profile of the survivor inversely correlated with bacterial load with no viable *C. difficile* detectable from day 10 indicating complete pathogen clearance (Figure 6D). The cecum at the endpoint showed marked reduced pathology compared to animals of both control groups that succumbed to infection (Figure 7A). Another of the CD0873-immunised animals showed only mild symptoms of CDI. This hamster may have also gone on to clear infection had it not been culled (Figure 6B). Conversely, animals immunised parenterally with toxoids showed no protection and remained fully colonised at levels indistinguishable from naïve animals (Figure 6) consistent with previous observations by Hong et al. (2017) [70].

The findings suggest that CD0873-specific sIgA antibodies bind CD0873 and coat the surface of vegetative cells of *C. difficile* which blocks their attachment to colonic epithelial cells, as supported by our adherence blocking results with Caco-2 cells (Figure 5B). In the survivor, we speculate the bacteria go on to multiply in the lumen instead and are then readily shed (Figure 6D). Indeed, the bacterial load in faeces of the survivor at day 4 exceeded the highest load of all other animals that succumbed to infection. The healthy body condition of the vaccinated hamster correlated with the lack of toxin-mediated damage to the cecum. Our data suggests that bacterial coating by sIgA may not only prevent toxins being brought into close proximity to epithelial cells by sterically hindering adhesion of *C. difficile* to enterocytes but may block toxin secretion itself and sIgA may additionally directly neutralise these toxins (Figure 7B). Furthermore, it appears that these vaccine-induced antibodies were not only antitoxic (be it directly or indirectly) but also antibacterial as seen by clearance of infection by day 10 (Figure 6D)

Mucosal sIgA is known for its ability to intercept infection by (a) preventing the adhesion of pathogens to the mucosal epithelium and (b) directly neutralising enterotoxins [56,71] or blocking their interaction with epithelial cells, as shown for cholera toxins [72]. Interestingly, intestinal sIgA generated in response to oral administration of the Dukoral vaccine, which contains recombinant cholera toxin B, is not only antitoxic but antibacterial and confers protection against both cholera and Enterotoxigenic *E. coli* [73]. Moreover, there have been several studies demonstrating a direct association between sIgA and enterotoxin TcdA of *C. difficile.* Firstly, Dallas and Rolfe (1998) reported that sIgA in human milk binds to TcdA. These workers proposed that this protease-resistant immunoglobulin naturally abundant in milk confers protection to breast-fed infants by preventing TcdA from binding intestinal epithelial cells [74]. Indeed, infants typically show high rates of colonisation with *C. difficile* but remain asymptomatic suggesting successful toxin interception or neutralisation. Secondly, oral immunisation of hamsters with spores of *Bacillus subtilis* engineered to express a repeat unit of the RBD of TcdA generated sIgA and IgG both of which were able to neutralise TcdA and TcdB with 75% protection against primary CDI and complete protection from re-challenge [75]. Crucially it was the mucosal antibodies that were found to correlate with the protection observed [70,75].

In evaluating the protective efficacy data for CD0873, there are several important points to note. Firstly, although a clear trend in protection was observed, non-significance was found. This was not surprising given that this was a pilot study using small *n* values Further studies with larger group sizes for statistical power are warranted. Secondly, variation was observed between hamsters, which is not uncommon and there are several contributing factors that apply specifically to this study. Enteric capsules and polymers available for rodents are less advanced than capsules used for humans with variation in delivery to the small intestine inevitable. For oral vaccines, more than one dose is often necessary to overcome tolerance (immunological unresponsiveness that can arise after oral administration of an antigen) yet how many doses of the three successfully localised to the small intestine is not known. Additionally, it is probable that the mode of challenge i.e., the choice of strain and size of inoculum used overwhelmed the immune system. Challenging with a lower number of spores that is more realistic of a natural infection may reveal that CD0873 is more protective than observed in this study.

To conclude, in addition to the attractive immunological properties of CD0873, we show that it can be successfully delivered in the form of lyophilised recombinant full-length protein since it is highly soluble. The propensity for lyophilised antigens to readily reconstitute in vivo is essential for structural reformation of immunological epitopes. Moreover, consistent detection of sIgA in every immunised animal in our immunogenicity study (3.3) suggests CD0873 has good bioavailability. In contrast, CDTcdB-RBD from the initial purification stage was insoluble and following lyophilisation, did not readily reconstitute in PBS. Its insolubility may largely explain the lack of immunogenicity observed for this antigen.

To immunise successfully against CDI, our work highlights the importance of oral delivery in generating intestinal sIgA to block colonisation as well as intercept and potentially neutralise the enterotoxins. In addition to CD0873, the inclusion of soluble immunogenic domains of the toxins are likely to enhance toxin neutralisation and it would also be beneficial to include an immunogenic domain of the binary toxin for further protection against hypervirulent isolates. Additional practical benefits of an oral *C. difficile* vaccine over injected vaccines are improved safety, easier manufacturing and administration with greater compliance particularly amongst the elderly, a key target population.

## Data Availability

Data is contained within the article or Appendix A.
